# The prevalence of idiopathic hirsutism and polycystic ovary syndrome in the Tehran Lipid and Glucose Study

**DOI:** 10.1186/1477-7827-9-144

**Published:** 2011-11-01

**Authors:** Fahimeh Ramezani Tehrani, Homeira Rashidi, Fereidoun Azizi

**Affiliations:** 1 Reproductive Endocrinology Research Center, Research Institute for Endocrine Sciences, Shahid Beheshti University of Medical Sciences, Tehran, Iran; 2 Diabetes Research Center, Ahvaz Jundishapur University of Medical Sciences, Tehran, Iran; 3 Endocrine Research Center, Research Institute for Endocrine Sciences, Shahid Beheshti University of Medical Sciences, Tehran, Iran

**Keywords:** Prevalence, Polycystic Ovary Syndrome (PCOS), NIH criteria, Tehran Lipid and Glucose Study (TLGS), Idiopathic Hirsutism (IH)

## Abstract

**Background:**

There is no clear and contemporaneous method for screening of idiopathic hirsutism (IH) and polycystic ovary syndrome (PCOS) at the community level and current estimates regarding their prevalence are limited. We aimed to ascertain the prevalence of IH and PCOS in a randomly selected sample of reproductive aged female participants of the Tehran Lipid and Glucose Study (TLGS).

**Methods:**

One thousand and two women, aged 18-45 years, were randomly selected from among reproductive aged women who participated in the TLGS. Those women with either hirsutism or menstrual dysfunction were assessed for biochemical hyperandrogenemia; whereas those participants with hirsutism per se were further assessed for subclinical menstrual dysfunction. PCOS were diagnosed using the National Institute of Health (NIH) criteria. IH was defined as hirsutism without clinical or sub clinical menstrual dysfunction or biochemical hyperandrogenemia (BH).

**Results:**

The mean ± SD of age of study population was 29.2 ± 8.7 years. Estimated prevalences of idiopathic hirsutism and pure menstrual dysfunction were 13.0% (95% CI: 10.9%-15.1%) and 1.5%(95% CI: 1.1%-1.9%), respectively. The prevalence of PCOS was 8.5% (95% CI: 6.8% - 10.2%); more than one third of these cases would possibly have remained undiagnosed or misdiagnosed, had we not assessed them for subclinical menstrual dysfunction or biochemical hyperandrogenemia.

**Conclusions:**

These data from a large representative and non selected population of women confirm the concept that IH and PCOS are the two most common gynecological endocrinopathies among reproductive aged women. The estimated prevalence of these conditions is highly influenced by their screening methods at the community level.

## Background

Hirsutism, the presence of terminal hairs in females in a male-like pattern, affects between 5% and 10% of women and is considered as idiopathic hirsutism (IH) with the presence of hirsutism and the absence of anovulation and/or hyperandrogenemia [1]. The definition of IH has varied significantly over the past three decades, along with changes in the definition of polycystic ovary syndrome (PCOS), however as IH is a diagnosis of exclusion, it is often difficult to fully differentiate these two disorders [2]. There are only a few community based studies documented that have estimated the prevalence of these two conditions [3-5].

The reported prevalence range of PCOS is between 2.2% to 26% [3,5-14] and it is estimated that approximately 5% to 20% of hirsute patients will have IH [1,2,15-17]. PCOS is a heterogeneous disorder and its influence on reproductive capability and metabolic disturbances including insulin resistance, type 2 diabetes mellitus, dyslipidemia and cardiovascular diseases is varied, according to its diagnostic criteria, race and recruitment of study subjects [18-22]; furthermore the metabolic and cardiovascular complications of IH have not been appropriately described [1,23].

Ethnic and racial variations strongly affect the clinical presentation of PCOS and IH [24-27]; the wide ranges of prevalence of these disorders can be further explained by differences in the recruitment process of the study population, controversy regarding its diagnostic criteria and the method used to define each criteria. The type of participants in each epidemiological study may potentially bias the result [12]; using the Rotterdam (Rott.) versus the NIH criteria, increases the PCOS prevalence by 1.5-2 times and undoubtedly this reduces the prevalence of IH [5,28-30].

Furthermore, relying only on clinical assessment for identification of hyperandrogenemia or menstrual dysfunction can easily overlook sub clinical oligo anovulation or hyperandrogenemia [16,31,32].

We aimed to identify the prevalence and clinical characteristics of PCOS and IH among a non-selected sample of reproductive aged women who participated in the TLGS.

## Methods

### Study subjects and the sampling method

The subjects of the present study were selected from the Tehran Lipid and Glucose Study (TLGS) [33]. TLGS is an ongoing prospective study in Tehran with the aim of determining the prevalence of non communicable disease risk factors; 15005 people aged 3 years and above, were selected from a geographically defined population using multistage cluster sampling method. There were 4290 women, aged 18-45 years who participated in TLGS; each woman was ranked according to a specific TLGS code, according to which, 1060 subjects were randomly selected using systematic random sampling method. We included all women regardless of hormonal therapy, including oral contraceptive pills or continuous progestin, glucocorticoid, or insulin sensitizer or anti androgen therapy. This is important because PCOS may predispose patients to the use of hormonal therapy and we would have underestimated the PCOS prevalence, had we not included these women. Menopausal women, those who had undergone hysterectomy or bilateral oophorectomy and pregnant women were excluded (n = 58).

A standard questionnaire including demographic variables and reproductive history, with emphasis on regularity of menstrual cycle, gynecological history, hyperandrogenic symptoms, family history of irregular menstrual cycle and hirsutism was completed, during face-to-face interviews and hirsutism was assessed using the modified Ferriman-Gallwey (mFG) scoring method [30] by a general practitioner who had been trained in a one month observer course at the PCOS clinic under supervision of a single endocrinologist (H.R.). Patients on hormonal therapy were questioned regarding their menstrual cyclicity before they started the medications and their hormonal assessment was excluded in the statistical analysis. Subjects with acne and or an initial mF-G score of over 3 and/or menstrual dysfunction defined as vaginal bleeding episodes at no less than 35-day intervals [32,33] were re-assessed by a single endocrinologist (H.R.).

An overnight fasting venous blood sample was obtained from those subjects with hirsutism defined as mFG >= 8/or menstrual dysfunction on the second or third day of their spontaneous or progesterone induced menstrual cycles (n = 260). In women with hirsutism only, serum was obtained on any one day between days 22-24 of the cycle for the measurement of progesterone (P4) to confirm ovulatory function (P4 level < 4 ng/ml, indicating anovulation). All sera were stored at -80°C until the time of measurements.

### Measurements

Dehydroepiandrosterone sulfate (DHEAS), 17-hydroxyprogesterone (17OH-P), Total testosterone (TT) and androstendion(A4) were measured by enzyme immunoassay (EIA), (Diagnostic biochem canada Co. Ontario, Canada). Sex Hormone Binding Globulin (SHBG) was measured by immunoenzymometric assay (IEMA), (Mercodia, Uppsala, Sweden). All ELISA tests were performed using the Sunrise ELISA reader (Tecan Co. Salzburg, Austria).

Luteinizing hormone (LH), Follicle stimulating hormone (FSH), Prolactin (PRL), and Thyroid stimulating hormone (TSH) were measured by the immunoradimetric assay (IRMA), (Izotop, Budapest, Hungary) using gamma counter(Wallac Wizard, Turku, Finland).

The free androgen index (FAI) was calculated using the formula [TT (nmol/L) × 100/SHBG (nmol/L)]. Intra-and inter-assay coefficients of variation for TT were 3.6% and 6.0%; for DHEAS: 1.9% and 3.2%; for 17 OH-P: 5.1% and 6.2%; for SHBG: 1.1% and 4.1%; for A4: 2.2% and 3.5%; for LH-2.8% and 5.6%; for FSH: 3.5% and 3.9%; for TSH: 1.9% 3.4%, and for PRL, they were 2.1% and 4.3%.

### Definitions

Clinical hyperandrogenism (CH) was defined by the presence of hirsutism (mF-G ≥8) [30], acne, or the presence of androgenic alopecia [2]. Clinical menstrual dysfunction was considered as vaginal bleeding episodes at no less than 35-day intervals [34,35] and sub-clinical menstrual dysfunction was defined as the presence of oligo-anovulation in eumenorrheic women (cycles 27-34 days in length). Oligo-anovulation was determined by measuring the serum progesterone level at any one day between days 20 to 24 of the cycle. If progesterone level was below 4 ng/mL, and the result was repeated in one more cycle, then the cycle was considered to be oligo-anovulatory [2]. Biochemical hyperandrogenemia (BH) was detected by FAI and/or DHEAS and/or A4 level, above the upper 95th percentile for the 40 women studied, who were not on any hormonal medication and had no clinical evidence of hyperandrogenism and menstrual dysfunction (total T = 0.89 ng/ml, A4 = 2.9 ng/ml, DHEAS = 179 μg/dL and FAI = 5.39). HA was determined as clinical hyperandrogenism (CH) and/or biochemical hyperandrogenemia (BH). Idiopathic hirsutism (IH) was defined as hirsutism without menstrual dysfunction and BH. PCOS was defined using NIH criteria as the combination of menstrual dysfunction and clinical hyperandrogenism and/or hyperandrogenemia (HA), after excluding hyperprolactinemia, thyroid dysfunction, nonclassic 21-hydroxylase deficiency (NC-CAH) and Cushing's syndrome.

### Statistical analysis

Continuous variables, checked for normality, using the one-sample Kolmogorov-Smirnoff test, are expressed as mean ± standard deviation and/or median and interquartile ranges, as appropriate. The categorical variables, expressed as percentages, were compared using the *x ^2 ^*test. Distributions between groups were compared using the Kruskal-Wallis test, followed by Mann-Whitney test with Bonferroni correction for pair wise comparison. Data analysis was performed using the SPSS 15.0 PC package (SPSS Inc., Chicago, IL).

### Details of Ethics Approval

The ethical review board of the Research Institute for Endocrine Sciences has approved the study proposal and informed consent was obtained from all subjects.

## Results

**Figure 1:**
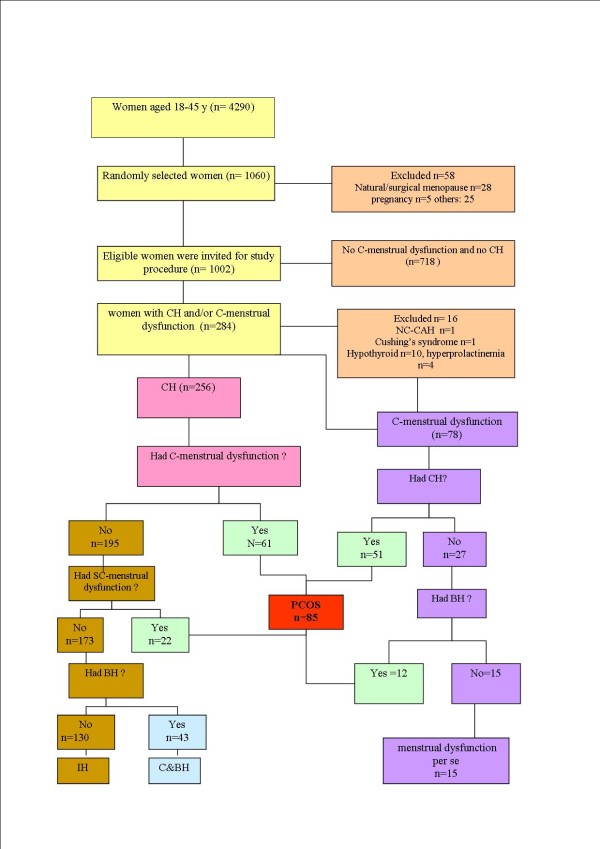
**Summary of study procedure**. CH, clinical hyperandrogenism; C-menstrual dysfunction, clinical menstrual dysfunction; SC-menstrual dysfunction; BH, biochemical hyperandrogenemia; IH, idiopathic hirsutism; PCOS, polycystic ovary syndrome using NIH definition; C&BH, clinical and biochemical hyperandrogenemia; NC-CAH, nonclassic 21-hydroxylase deficiency. *The serum concentration of mid luteal phase progesterone of 195 out of 205 women was measured.

The study procedure is summarized in Figure 1. A sample of 1060 women was randomly selected from among 4290 women, aged 18-45 years, who participated in TLGS. The mean age of study population was 29.2 years (age range: 18-45 years). Of the 1002 eligible women included in the study, 30.0% were overweight (BMI, 25.0-29.9 kg/m^2^), and 20.5% were obese (BMI≥30.0 kg/m^2^).

Of a total of 1002 women, 718 women were found to have no hirsutism or clinical menstrual dysfunction based on their history and physical examination; they were not further evaluated and considered as normal eumenorrheic non hirsute women. Of 256 women with hirsutism, 246 ones completed the study procedure, of these 246, 43 women (4.3%) had both BH and CH, without PCOS and 130 had IH; the estimated prevalence was 13.0% (95% CI: 10.9%-15.1%). The prevalence of subclinical menstrual dysfunction among women with hirsutism was 11.3%(22/195). There were three cases of acne-per se but all of them had biochemical hyperandrogenemia. Of 78 women with clinical menstrual dysfunction who completed the study procedure, 15 subjects had menstrual dysfunction per se, while the prevalence of BH among non hirsute women with menstrual dysfunction was 44.4% (12/27); there were no significant differences in reproductive history and results of the first assessment for androgen excess symptoms between those who completed the study and those who did not (data was not shown).

The estimated prevalence of PCOS was 8.5%(95% CI: 6.8%-10.2%) using the NIH definition. Of the 85 women with PCOS, 51 had both clinical hirsutism and menstrual dysfunction. There were 12 PCOS women with only clinical hirsutism and 22 women with clinical menstrual dysfunction per se and both groups would possibly have remained undiagnosed, had we not assessed either the serum concentrations of androgens in non hirsute women with menstrual dysfunction or the mid luteal phase of progesterone of hirsute women.

Out of 21 women with a screening 17-OHP levels >6 nmol/L, there was one subject with NCCAH using the 17-HP response to ACTH stimulation test. Of twenty women who had features suggestive of Cushing's syndrome, one had a positive overnight low dose Dexamethasone suppression test. No suspicious case of an androgen secreting tumor was seen among study participants.

The basic, reproductive, metabolic and hormonal profiles of those who had various phenotypes of menstrual dysfunction and androgen excess disorders in comparison to normal women are shown in table 1. Compared to healthy subjects, the familial history of hirsutism was more prevalent among all other phenotypes but only women with IH had a significant higher BMI in comparison to the normal group. While serum concentrations of TT did not differ in women with the menstrual dysfunction per se in comparison to PCOS participants, their SHBG was significantly higher than the latter (Table 1).

**Table 1:** Characteristics of various phenotypes of androgen excess and/or anovulation

Group	Group1 (PCOS) N = 85	Group 2 menstrual dysfunction per se N = 15	Group 3 (CH+BH) N = 43	Group 4 (IH) N = 130	Group 5 (Normal) N = 718
**Age**	a 27.0 (21.0-35.0)	29.0 (20.0-34.5)	30.0 (23.0-35.0)	33.0 (26.0-38.0)	29.0 (21.0-36.0)
**Age at menarche**	13.0 (12.0-13.0)	13.0 (12.5-13.5)	13.0 (13.0-14.0)	13.0 (12.0-14.0)	13.0 (13.0-14.0)
**Women with history of primary Infertility %**	b 8.2	5.7	3.7	2.2	1.7
**Women with family history of hirsutism %**	b 58.8	39.6	b 40.7	b 49.6	24.9
**Women with family history of anovulation %**	b 32.9	30.2	29.6	24.1	17.0
**Women with family history of infertility %**	b 9.4	3.8	4.8	2.9	2.9
**Women with history of abortion %**	14.1	18.9	11.1	17.5	17.3
**No. Pregnancy**	1.0 (0-2.0)	1 (0-2.0)	0 (0-2)	2.0 (1.0-3.0)	1 (0-2)
**BMI (kg/m2)**	26.2 (22.3-30.5)	26.2 (22.4-29.2)	24.1 (22.1-27.9)	b 26.7 (23.7-29.7)	24.5 (21.2-28.7)
**Waist circumference (cm)**	85.0 (78.0-98.5)	83.0 (75.5-92.5)	81.0 (74.0-92.0)	88.0 (79.5-98.5)	82.0 (73.0-91.0)
**Hip circumference (cm)**	104.0 (97.0-110.5)	101.0 (96-109.5)	99.0 (94.0-110.0)	b 105.0 (100.0-110.5)	101.0 (95.0-107.8)
**Wrist circumference (cm)**	15.5 (15.0-16.5)	15.5 (15.0-16.2)	15.2 (14.5-16.0)	15.8 (15.2-16.5)	15.5 (15.0-16.2)
**Systolic Blood Pressure (mmHg)**	102.5 (98.0-111.3)	100.0 (90.3-112.3)	106.0 (98.0-112.3)	106.5 (98.3-116.8)	106.0 (98.0-114.0)
**Diastolic Blood Pressure (mmHg)**	70.0 (65.0-75.0)	70.0 (68.8-81.3)	72.0 (68.8-81.3)	71.5 (65.0-77.0)	71.0 (64.0-76.0)
**FBS**	87.0 (81.5-91.5)	101.0 (96.0-109.5)	86.0 (81.0-89.0)	87.0 (81.3-95.0)	86.0 (81.0-92.0)
**TT**	a 0.5 (0.3-0.7)	0.5 (0.4-0.8)	c 0.4 (0.3-0.8)	d 0.3 (0.2-0.4)	0.4 (0.2-0.6)
**SHBG**	b, e 37.0 (27.0-43.0)	b, f 58.0 (46.0-106.0)	36.0 (25.0-41.0)	b, d 38.0 (28.0-45.0)	45.5 (39.0-54.8)
**FAI**	a, b, e 3.4 (2.2-3.8)	f 2.1 (2.0-4.4)	c 5.1 (3.2-7.7)	2.4 (1.9-3.6)	2.4 (1.5-4.2)
**DHEAS**	119.0 (77.0-162.3)	122.0 (85.7-139.2)	c 180.0 (123.0-221.8)	96.0 (64.0-130.0)	124.0 (75.5-145.3)
**A4**	b 1.4 (0.6-2.3)	1.1 (0.3-1.4)	1.5 (0.9-2.7)	1.1 (0.5-1.6)	0.8 (0.5-1.7)

PCOS, polycystic ovary syndrome using NIH definition; CH, clinical hyperandrogenism; BH, biochemical hyperandrogenemia; IH, idiopathic hirsutism; Normal group, eumenorrheic without hirsutism. BMI, body mass index; DHEAS, Dehydroepiandrosterone sulfate; Total T, Total testosterone; A4, Androstendion; SHBG, Sex Hormone Binding Globulin; FAI, Free androgen index

Values are given as median (Inter quartile range), a = Group 1 vs. Group 4; b = vs. normal group, p < 0.001, c = Group 3 vs. Group 4, p < 0.001; d = Group 2 vs. Group 4, p < 0.001; e = Group1 vs. Group 2, p < 0.001; f = Group 2 vs. Group 3, p < 0.001

The hormonal profile of women on hormonal therapy (n = 31) were excluded from statistical analysis.

## Discussion

The present study demonstrated that the prevalences of IH and PCOS (using the NIH definition) in a sample of reproductive aged women who participated in Tehran Lipid and Glucose Study were 13.0% (95% CI: 10.9%-15.1%) and 8.5%(95% CI: 6.8%-10.2%), respectively. More than one third of affected women (34/85) would have been possibly misclassified, had we not assessed participants for subclinical menstrual dysfunction or biochemical hyperandrogenemia.

In spite of the potential public health impact of the PCOS there are relatively few studies that estimate its prevalence at the community levels and most studies available have relied upon convenience samples [3,11-13] that potentially bias their results. The reported prevalence range of PCOS is between 2.2% to 26% [3,5-13]. In addition to different recruitment processes of the study population, selection bias, ethnic and racial variation, the wide range of estimated PCOS prevalence can be explained by the criteria used for its definition and the screening methods used to identify each criteria [4,6,8,36,37]; considering the Rotterdam versus NIH criteria increase the PCOS prevalence by 2 times [5,21,28,29,38]. Our study is comparable with those few community based studies that provide an estimation regarding the PCOS under NIH criteria [4,5,28]. Similar to our estimation, the prevalence of PCOS using NIH definition was reported to be 8.7% among 978 women, who were recruited in a retrospective birth cohort study in South Australia [4], however our estimation is much higher than that implied by Kumarapeli et al in a non selected population in Sri Lanka [28]; the racial variation in hyperandrogenic manifestation of PCOS [39,40]and the lower degree of hirsutism among indigenous Sri Lankans might explain the difference observed [28].

There is no clear and contemporaneous recognition of each PCOS criteria; this could highly influence the PCOS prevalence estimated. In our study, clinical hyperandrogenism was determined as mF-G ≥8 and menstrual dysfunction was defined as vaginal bleeding episodes at no less than 35-day intervals or P4 level < 4 ng/ml in the mid luteal phase; some other investigators have used different cut of points for these definitions, e.g. Asuncion et al. used six or fewer menstrual cycles in a year as the definition for menstrual dysfunction [3], the clinical hyperandrogenism was defined as mF-G ≥6 by Knochenhauer et al. [10]and as mF-G ≥7 by DeUgarte et al. [26].

There is no agreement on widespread screening of PCOS criteria; it is not clear whether or not biochemical investigations should be evaluated for those non hirsute women with regular and predictable menstrual cycles or should the serum concentration of all types of androgens be universally measured to identify those women with sub clinical hyperandrogenemia. The study protocol for screening of PCOS criteria at the community level has a great impact on the PCOS prevalence estimated. We did not measure the mid luteal phase serum progesterone or androgens for those women has neither hirsutism nor clinical menstrual dysfunction in their first clinical assessment. It has been shown that 3.7% of eumenorrheic, non hirsute women have oligo-ovulatory cycles diagnosed by serum concentration of progesterone [31]; therefore the number of women with menstrual dysfunction in our study might have been increased from 78 to 105 [(78 + (718 × 3.7%)] had we assessed mid luteal phase serum progesterone of all participants. To verify hyperandrogenemia, we measured serum concentration of all types of androgens among those women with either clinical menstrual dysfunction or hirsutism but it has been shown 5.4% of women with BH would have remain undiagnosed had we not assessed the serum concentrations of androgens, regardless of regularity of their menstrual cycles [5].

The reported prevalence of idiopathic hirsutism varies between 5-20% [1,5,16,41-43], being 13.0%(12.8%-13.2%) in the present study. There are significant racial variation in prevalence of IH; East Asians are typically less hairy than Euro-Americans, which may be explained by low levels of 5a-reductase activity in the skin of those women [27]. It has been reported that 50-70% of all hirsute women demonstrate regular menses, suggestive of IH[1]; in our study 80%(205/256) of hirsute women had regular and predictable menses; among them the anovulatory cycle of 22 women(11.3%) would possibly have been overlooked had we did not measured the mid luteal phase serum concentration of progesterone. In the present study, 130 women had IH; these women cannot be entirely excluded from the diagnosis of PCOS and undoubtedly using the Rotterdam criteria will further reduce this number [2]. However the appropriateness of applying the Rotterdam criteria have been criticized and the additional phenotypes which are diagnosed as PCOS by this criteria have given rise to considerable debate in recent literature [44]; it is essential to clearly identify the impact of adding these phenotypes in research, clinical practice and also quality of life of those women who may have been prematurely labeled as having PCOS.

We excluded women with natural or surgical menopause; however it is unlikely that our results were influenced by not including those women. While the incidence of uterine leiomyomata was 65% higher among women with PCOS, than in those without the condition [45] and women with PCOS were more often hysterectomized [46], however the majorities of hysterectomies are conducted among women above our age limit(45 y). It has been shown that the natural age at menopause in PCOS women is higher compared with normo-ovulatory controls [47]. Since the mean age at natural menopause was 50.4 years (S.D. = 4.3) for the general population of Iranian women in a large national survey [48],(much higher than our age limit), our estimation is possibly not affected by excluding the menopausal women. With ageing the clinical manifestations of PCOS change and its reproductive abnormalities decrease along with increase of its metabolic manifestations; as a result our estimations are not generalizable to postmenopausal women.

The main strength of the present study is its methodology as it is a community-based prevalence study on a primarily ethnically homogeneous population with a response rate of more than 90%. There few communities based PCOS prevalence studies documented, because of logistical problems in performing such investigations in a large sample in a community setting. The amount of intra-assay variability in our data is also likely to be minimal because all the laboratory measurements were done at the same laboratory by the same person.

Our study does have some limitations; we did not consider vaginal ultrasonography and as a result could not provide estimations using any definition other than the NIH one. If the Rotterdam criteria were used and polycystic ovaries had been seen on scan, some women that were classified as "IH" would possibly reclassified as "PCOS" and some participants that previously belonged to either the "pure hyperandrogenism" or "pure menstrual dysfunction" groups, would have been diagnosed as PCOS using Rotterdam criteria. Our results may be underestimates as the androgens and mid luteal serum progesterone measurements were not evaluated for those non hirsute women with regular and predictable menstrual cycles; however it has been shown that only 3.7% of eumenorrheic women had anovulation [31]. Hence, it is likely that only a few women with PCOS under NIH criteria were present in the population of eumenorrheic non hirsute women.

## Conclusion

These data from a large community based study confirm the concept that PCOS and IH are the two most common gynecological endocrinopathies among reproductive aged women and the estimated prevalence of these conditions is highly influenced by their screening methods for identification of the milder and subclinical phenotypes at the community level. A universal strategy for screening of PCOS at the community level needs to be developed for improving the comparability and potentially the value of published research and the widespread screening of mild and sub clinical phenotypes of PCOS have to be justified.

## Competing interests

The authors declare that they have no competing interests.

## Authors' contributions

FRT participated in the design of the study, performed the statistical analysis and drafted the manuscript. HR participated in the coordination of the study and helped to draft the manuscript. FA participated in the design of the study and helped to draft the manuscript. All authors read and approved the final manuscript.
